# Testing for heterogeneity among the components of a binary composite outcome in a clinical trial

**DOI:** 10.1186/1471-2288-10-49

**Published:** 2010-06-07

**Authors:** Janice Pogue, Lehana Thabane, PJ Devereaux, Salim Yusuf

**Affiliations:** 1 Department of Clinical Epidemiology and Biostatistics, McMaster University, Hamilton, Ontario, Canada; 2 Faculty of Health Sciences, McMaster University, Hamilton, Ontario, Canada

## Abstract

**Background:**

Investigators designing clinical trials often use composite outcomes to overcome many statistical issues. Trialists want to maximize power to show a statistically significant treatment effect and avoid inflation of Type I error rate due to evaluation of multiple individual clinical outcomes. However, if the treatment effect is not similar among the components of this composite outcome, we are left not knowing how to interpret the treatment effect on the composite itself. Given significant heterogeneity among these components, a composite outcome may be judged as being invalid or un-interpretable for estimation of the treatment effect. This paper compares the power of different tests to detect heterogeneity of treatment effect across components of a composite binary outcome.

**Methods:**

Simulations were done comparing four different models commonly used to analyze correlated binary data. These models included: logistic regression for ignoring correlation, logistic regression weighted by the intra cluster correlation coefficient, population average logistic regression using generalized estimating equations (GEE), and random effects logistic regression.

**Results:**

We found that the population average model based on generalized estimating equations (GEE) had the greatest power across most scenarios. Adequate power to detect possible composite heterogeneity or variation between treatment effects of individual components of a composite outcome was seen when the power for detecting the main study treatment effect for the composite outcome was also reasonably high.

**Conclusions:**

It is recommended that authors report tests of composite heterogeneity for composite outcomes and that this accompany the publication of the statistically significant results of the main effect on the composite along with individual components of composite outcomes.

## Background

Composite outcomes can often be difficult to interpret, especially when the treatment effects on some of its components individually show differences in magnitude or even in direction. For example, in a trial of localized intracoronary gamma-radiation therapy versus placebo [1] the primary composite outcome of death, myocardial infarction, or revascularization of target lesion showed an overall benefit of gamma-radiation compared to placebo (24.4% vs 42.1%, p = 0.02); however, myocardial infarction individually had a non-significant effect in the opposite direction (9.9% vs. 4.1%, p = 0.09). Many authors have expressed concerns regarding interpretation of a treatment effect for a composite outcome when it appears that there is heterogeneity in the treatment effect across the composite components [2-4]. How then can we best determine the existence of important composite heterogeneity in treatment effect among the individual components of a composite outcome?

A composite outcome is defined as having occurred if one of a group of outcomes occurs. The main treatment effect is defined as the absolute or relative difference between treatment and control in the proportions of participants who have at least one component of the composite. The problems with interpreting composite outcomes are well known. The treatment effect observed on the components may go in opposite directions and reduce the power of the trial [5,6]. The components may not have similar importance or frequency to one another [2-4,7]. These issues make composite outcomes difficult to interpret in many trials.

Despite difficulties with interpretation, trialists are unlikely to abandon composite outcomes. Trials in cardiovascular disease commonly use composite endpoints as their primary outcome [8] and there are efforts in many other areas of research to follow suit. Many authors have expressed the need to use composite outcomes to increase the feasibility of conducting clinical trials research in their areas including: cardiology [9,10], HIV/AIDS [11], organ transplantation [12], psychiatric disorders [13], adverse event reporting [14], and obstetrics and gynecology [15]. The reasons for use of composite outcomes are well documented and include: reduced sample size due to increased outcome rates, the ability to answer important questions quickly, capturing the multi-dimensional nature of disease, seeking a better understanding of total disease burden, the inability to select the most important of many outcomes, concerns with multiplicity for testing many outcomes, and addressing competing risks.

Various approaches have been suggested for the analysis and interpretation of composite outcomes. For example, a multivariate global test across all the components could be used to look for simultaneous demonstrated benefit; but readers may find it difficult to interpret such a result [16,17]. Alternatively, if the composite shows a statistically significant treatment effect, the component specific tests can be performed using a closed test procedure. Many authors recommend that each component of the composite should be defined as secondary outcomes for the trial [6]. However, it is doubtful that there would be sufficient power to detect effects on the individual components for the very reason that the composite outcome was chosen (i.e. there are too few events for each outcome). Individual tests on each component would also inflate the overall Type I error rate for the study. Berger [18] has suggested the use of informative preserving composite endpoints and the use of omnibus test functions. However, trialists have rarely utilized this procedure. Finally, another method would involve analysis of the weighted components of the composite. Although many different weighting schemes have been suggested [6,9,19,20], these methods are not in common use by trialists [5]. Further, weighting systems can introduce their own set of problems with interpretation, due to the perceived subjectivity of the weights.

Composites may be used either under the assumption of homogeneity of treatment effect across components or to summarize a risk-benefit profile of an intervention. In this manuscript we address the former use, where the best knowledge of the disease being studied points to a likely similarity of treatment effect on all component outcomes, based on known physiological pathways and theoretical models. While the treatment effect is assumed to be similar across each of the components in terms of direction, it is recognized that the magnitude may differ [2,5]. Many authors recommend reviewing suspected treatment homogeneity through visual inspection of the direction of relative risk estimates for individual components of the composite in a trial [2,7]. However, it is possible to test for heterogeneity of these treatment effects across components directly using standard methods for correlated binary data. If significant heterogeneity is found then the composite outcome may be invalidated or inappropriate for use. If not, we may have more confidence in the composite outcome, viewing it as meaningful, interpretable to represent treatment effect as a whole, and likely free from evidence of heterogeneity. However, tests for heterogeneity have been shown to lack power in meta-analyses and subgroup analyses [21]. The purpose of this paper is to compare the power of different tests to detect heterogeneity of treatment effect across components of composite binary outcomes. We then explore the usefulness of such tests for detecting composite heterogeneity when the power is high for the treatment comparison on the composite outcome as a whole.

## Methods

### A. Methods for analysis of correlated binary outcomes

Participants in a trial who are followed beyond their first outcome may experience more than one component of the composite primary outcome. For example, for a trial with the primary outcome of myocardial infarction, stroke or cardiovascular death, a participant may experience a stroke and then die a cardiovascular death. Thus there is a repeated measurement of the different component outcomes for each individual. This binary data then has an intra cluster correlation due to repeated outcomes on the same individuals.

All models used contain parameters that estimate the treatment effect, the specific individual outcome component in the composite outcome, and the interaction of these two factors. These are presented for the jth treatment group, the kth component of the composite component outcome, and the ith participant in the trial. The test of the interaction term will allow detection of possible heterogeneity or difference in the study treatment effect across the composite components.

The following models will be studied using SAS 9.1 [22] as presented in Shoukri and Chaudhary [23]:

#### Model 1 Logistic regression ignoring correlation

It is possible that the intra cluster correlation seen among outcomes in typical cardiovascular trials is too small to make a difference to this analysis of composite homogeneity. We will fit a simple logistic regression to test this hypothesis (implemented in SAS using *proc logistic *[22]). The model fit will be: *Logit*(*y*_*ijk*_) = *β*_*0 *_+ *β*_*1*_*x*_*1 *_+ *β*_*2*_*x*_*2 *_+ *β*_*3*_*x*_*3 *_+ *ε*_*ijk*_

Here y_ijk _is a binary response representing whether an event (i.e. one of the components of a composite outcome) has occurred (coded 1) or not (coded 0). The fixed factors for all participants are the intercept *β*_0_, treatment effect *β*_1_, composite outcome component *β*_2_, and interaction of treatment and outcome *β*_3_. With more than two component outcomes to the composite, there would be additional regression coefficients for each additional component and an additional term for its interaction with treatment. The error term *ε*_*ijk *_here does not take into account the correlation of composite outcome components within each individual. Therefore, the fitted regression coefficients are:

For example, the following matrices display the outcomes status (**Y**) and independent variables (**X**) for the first two participants in our simulation. Since our composite outcomes has two components, the vector Y has two rows for each participant with the first containing the outcome status (0,1) for the first component and the second row for the outcome on the second component. Both of the following participants have experienced a composite outcome. Participant 1 experienced both components of the composite outcome and participant 2 experienced only the second component.

For this and all subsequent models, the test for heterogeneity will test whether *β*_*3 *_is significantly different from zero at p < 0.05 level.

#### Model 2 Weighted logistic regression

Simple methods for the analysis of binary correlated data have been suggested using weighted logistic regression. Donald and Donner [24] proposed a weighting based directly on the intra cluster correlation (*ρ*) calculated for the trial overall and Rao and Scott [25] base the weights on the variance inflation factor (*υ*) estimated per treatment group (*proc logistic *[22] with weights *ρ *or *υ*). Note that a single weight may not be appropriate with more than two components to the composite outcome. The fitted regression coefficients are:

#### Model 3 Population average logistic models (GEE)

Here treatment and outcome component effects are estimated at the margin by averaging across individuals. The generalized estimating equations (GEE) methods will be used, which treats the correlation among individuals as a nuisance factor. Correlation between outcomes of individuals is modeled through a working correlation matrix and adjustments for misspecification are made using the sandwich variance formula [26]. The covariance matrix will be unstructured to allow for different variances for each composite component (*proc genmod *[22]). The model is: where *μ*_*ijk *_= E(*y*_*ijk *_), the marginal expectation and the β*'s estimate the population average response parameters.

#### Model 4 Random effects logistic models

This model incorporates a term for the individual in the analysis and allows the intercept to vary across individuals. Individuals are considered to be randomly selected from a population that has a normally distributed intercept component [27]. The model is

*Logit(E[y*_*ijk*_|*γ*_*k*_]) = *β*_*0 *_+ *β*_*1*_*x*_*1 *_+ *β*_*2*_*x*_*2 *_+ *β*_*3*_*x*_*3 *_+ *γ*_*i *_+ *ϕ*_*ijk *_where *γ*_*i  *_is the random effect of participant with composite outcome component clustered within individual and *ϕ*_*ijk *_is the error term (*proc glimmix *[22]). The covariance matrix will be unstructured, or determined by the random effect.

### B. Simulation data

The purpose of this simulation was to examine the power to detect heterogeneity among the components of a composite outcome for a well-designed trial. We began with a study design that had good power to detect a modestly estimated main treatment effect on the odds ratio (OR). Such a design was chosen since it is unlikely that a composite outcome heterogeneity test would be performed if the main treatment effect were not statistically significant. The total study sample size was 2000 for a two-arm trial with equal allocation to each treatment group, and a 50% composite outcome event rate in the control group. This was calculated using a continuity corrected chi-square test of equal proportion with two-sided type I error rate of 0.05. There was 88% to detect a 25% reduction in the OR and 97% power for a 30% OR. A composite with two components was simulated with a correlation between the two components of *ρ *= 0.10 (estimated using cardiovascular outcomes from the HOPE trial [28], unpublished data). Simulations were run with 10,000 iterations and we recorded both power for the test of treatment effect on the composite outcome and for the heterogeneity of treatment across the composite components for each model. We examined the power for these tests by varying the following:

a) Degree of treatment heterogeneity of the composite components: The odds ratio of the first component (OR_1_) was kept constant, while the second component odds ratio (OR_2_) was varied to simulate composite heterogeneity. Low heterogeneity is demonstrated by both OR's showing the same direction of treatment effect, moderate is indicated by a neutral effect in one component, and large is seen where the OR's have opposite patterns of risk.

b) Balance of the components: Simulations included cases where the components occurred equally (1:1) or unequally. For the unequal case, the composite outcome contained one component that occurred three or five times more often than the other.

Multivariate binary correlated data was generated using the method described in Park et al. [29]. Sums of independent Poisson random variables were generated which share components such that the resulting sums are multiple correlated Poisson variables. Indicator functions were used to transform these variables into correlated binary data with the desired correlational structure.

## Results

**Figure 1:**
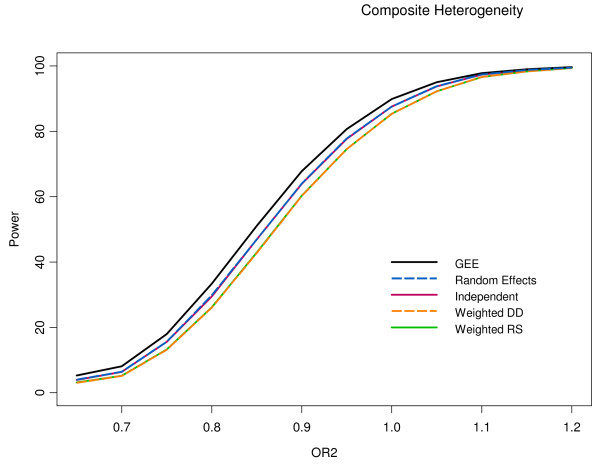
**Power for composite outcome heterogeneity by model as a function of treatment effect for the second component**. Note that power curves for both weighted models completely overlap in this figure. Independent and Random Effects line also overlap to a large degree.

As expected the power to detect heterogeneity among the composite outcome components increased as the difference between the two component odd ratios became larger (see Table 1 and Figure 1). The Population Average logistic regression had the greatest power across all levels of composite heterogeneity. The next largest power was seen in both the independent and random effects logistic regressions. Lastly, the weighted logistic regression displayed the least power for this test. It should also be noted that the population average model had a type I error rate of 0.053 for the case of no composite heterogeneity, exceeding chance level of 0.05.

**Table 1:** Power to detect heterogeneity between the two components of a composite outcome by degree of heterogeneity (equal balance among components) with OR_1 _= 0.65

Hetero- geneity	OR_2_	Composite Overall OR	Weighted DD	Weighted RS	Independent	Random Effects	GEE
None	0.65	0.65	3.0	3.2	3.9	4.0	5.3
	0.70	0.67	5.1	5.2	6.3	6.4	8.1
Low	0.75	0.70	13.1	13.2	15.6	15.6	17.9
	0.80	0.72	26.0	26.2	29.5	29.8	33.4
Moderate	0.85	0.75	42.7	42.9	46.9	46.9	51.1
	0.90	0.78	60.2	60.3	63.9	64.0	67.8
	0.95	0.80	74.6	74.6	77.7	77.8	80.7
	1.00	0.83	85.3	85.4	87.6	87.5	89.9
High	1.05	0.85	92.2	92.3	93.8	93.8	95.0
	1.10	0.88	96.6	96.7	97.4	97.4	97.8
	1.15	0.91	98.4	98.4	98.8	98.8	99.0
	1.20	0.93	99.4	99.4	99.6	99.5	99.7

When imbalance existed between the frequencies of the two components the power to demonstrate heterogeneity decreased as this imbalance increased (see table 2). This power was greater when the component displaying moderate treatment heterogeneity was also the less frequent of the two components. Note again that population average logistic model had the greatest power, except for the single case of 1:5 imbalances, where the component with the larger OR was the most frequent. For this case only, the weighted logistic regression had the greatest power and the population average logistic regression had the second greatest power.

**Table 2:** Power for detecting heterogeneity of treatment effect by varying degrees of balance among the components of the composite for a moderate heterogeneity pattern OR_1_, OR_2_= (0.65, 1.00) and ratio (p_1_:p_2_) of occurrence of components 1 and 2.

Balance (p_1_:p_2_)	Weighted DD	Weighted RS	Independent	Random Effects	GEE
1:1	85.3	85.8	88.1	88.2	90.0
1:3	77.0	77.1	75.4	75.4	78.7
1:5	65.0	65.0	59.4	59.4	62.8
3:1	79.1	79.1	79.5	79.9	82.3
5:1	70.3	70.3	68.2	68.6	71.1

**Figure 2:**
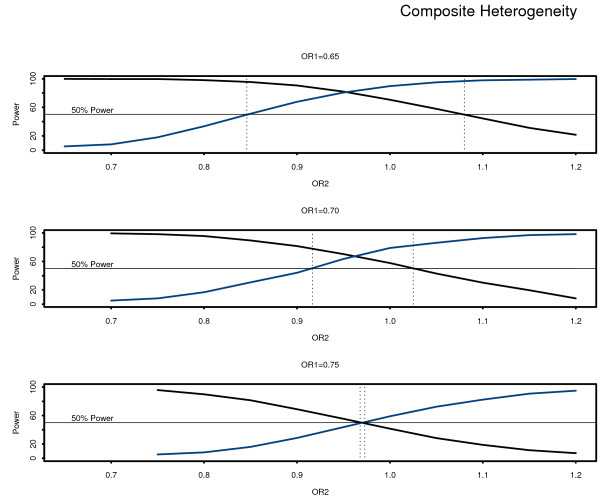
**The power for the main effect of treatment (black line) and the power for the test of heterogeneity of the composite components (blue line) by degree of composite heterogeneity**.

Table 3 and Figure 2 show the relationship between power for the test of treatment on the composite outcome as a whole and power to detect treatment heterogeneity among it components, using the population average model. Both the effect size of the composite outcome and the degree of composite heterogeneity are varied to show the relationship in power for both tests. The region in bold for this table indicates the conditions when both tests show greater than 50% power, over various combinations of the two odd ratios for each component. This is illustrated in Figure 2, where the region between the vertical dotted lines indicates the range where both the test of the composite outcome and the test for composite heterogeneity are both have 50% power or greater. When the odds ratio for the most effective component is 0.75, this region is the narrowest.

**Table 3:** Comparison of power for the main treatment effect with power for interaction test, using the population average model (GEE)

	OR_1 _= 0.65	OR_1 _= 0.65	OR_1 _= 0.70	OR_1 _= 0.70	OR_1 _= 0.75	OR_1 _= 0.75
**OR**_**2**_	Treatment Effect	Heterogeneity Test	Treatment Effect	Heterogeneity Test	Treatment Effect	Heterogeneity Test
0.65	>99.9	5.3	-	-	-	-
0.70	99.9	8.1	99.4	5.0	-	-
0.75	99.6	17.9	98.2	8.3	95.7	5.5
0.80	98.2	33.4	95.7	16.7	89.8	8.3
0.85	***95.5***	***51.1***	89.5	30.5	81.5	16.0
0.90	***90.7***	***67.8***	81.6	44.1	68.7	28.6
0.95	***82.2***	***80.7***	***70.4***	***63.5***	55.5	43.7
1.00	***70.7***	***89.9***	***57.8***	***78.8***	41.5	58.9
1.05	***57.7***	***95.0***	42.9	86.3	28.2	72.4
1.10	44.6	97.8	30.2	92.8	18.9	82.4
1.15	31.3	99.0	19.6	96.8	11.3	90.5
1.20	21.5	99.7	8.1	98.3	7.2	94.8

## Discussion

These simulations demonstrate that generally the population average (GEE) model has the greatest power to detect composite outcome treatment heterogeneity, of the four methods investigated. This is further supported by the conclusion that population average models (GEE) are the more powerful test among possible methods for analyzing cluster randomized trials data [30]. It should be noted that the GEE and random effects models do not estimate the same parameters, since GEE is a marginal model and the random effects allows the estimation of individual effects. For effect estimation the GEE models are known to bias model parameter estimates towards the null, but at the same time have smaller parameter standard deviations compared to random effects models [31]. Since the focus for this application is on the test statistics itself, rather than estimation, it seems reasonable that the population average model would have the greatest power. We found only one exception to this conclusion. When there was a large imbalance between the two composite components, where the most frequent of these had the smaller treatment effect, the weighted regression model had higher power, with the population average (GEE) model being second. We should also consider the fact that the GEE model was somewhat liberal in its type I error rate for the case of no composite outcome heterogeneity.

Even small amounts of component heterogeneity, can reduce study power to detect a treatment effect for the composite outcome. However, we did find regions where the power for both tests for the composite outcome and composite heterogeneity were greater than 50%. This indicates a range of results where tests for composite heterogeneity would be useful. One may only want to perform a test of composite outcome heterogeneity when the main effect is statistically significant but regardless of the statistical significance of the composite outcome, test for composite heterogeneity may provide insight into the differing mechanisms for each component outcome. This information could then aid in the design of future trials. However, for the current trial, the presence of composite heterogeneity should never lead researchers to assume that the composite outcome as a whole would have been statistically significant if only the mix of components were slightly altered.

The use of models for correlated binary data to explore composite outcome heterogeneity has some important advantages. It can easily be implemented in common statistical software packages using currently available repeated/recurrent outcomes methods. The methodology suggested in this manuscript can be generalized to other outcomes types in addition to binary, including continuous outcomes, time to first event and time to recurrent events. Given the ease of implementation and application to a variety of outcome types, trialists may be encouraged to address the issue of potential composite heterogeneity more often and more directly in the presentation of trial results.

There are limitations to the results presented here. We have not explored differing event rates, component correlations, extreme imbalance in component ratios, and the effects of more than two composite components. This area will require more research and such simulations could be a productive exercise when designing a randomized clinical trial. The methods presented would not be appropriate to use when the composite components are expected to show differing treatment directions, as in a risk-benefit composite outcome. Lastly, failure to detect statistically significant composite heterogeneity may be a result of lower power, rather than true treatment homogeneity across the composite components. Trialists would be wise to consider the power to detect composite heterogeneity in the design of trials with composite outcomes.

The methods of exploring composite outcome heterogeneity directly, using the tests described here, may partially address the concerns raised about using composite outcomes in many fields. When reporting trial results, it would seem reasonable to expect to see such a test for composite heterogeneity presented along side a statistically significant treatment effect test for the composite outcome.

## Conclusions

We compared the power of different tests to detect composite heterogeneity for treatment effect across components of a composite binary outcome. Simulations were done comparing four different models commonly used to analyze correlated binary data. The results of these simulations are quite clear. Generally, GEE model should be chosen for investigating possible heterogeneity among the components of a binary composite outcome, since it demonstrated the greatest power. This is particularly true when the power for the test of treatment effect on the composite outcome as a whole was also reasonably high. It is recommended that tests of composite heterogeneity for composite outcomes accompany the publication of the results for statistically significant composite outcomes along with individual components of composite outcomes. Further simulations are still required to explore the impact on power of differing event rates, component correlations, extreme imbalance in component ratios, and the effects of more than two composite components.

## Competing interests

The authors declare that they have no competing interests.

## Authors' contributions

JP conceived of applying the methods presented to analysis of binary composite outcomes, performed the simulations, and produced the figures. All authors helped define the conditions of the simulations and participated in drafting the manuscript. All authors have read and approved the final manuscript.

## Pre-publication history

The pre-publication history for this paper can be accessed here:

http://www.biomedcentral.com/1471-2288/10/49/prepub
